# Ethical issues surrounding breast cancer screening in Brazil

**DOI:** 10.6061/clinics/2019/e1573

**Published:** 2019-11-04

**Authors:** Rodrigo Gonçalves, José Maria Soares-Jr, Edmund Chada Baracat, José Roberto Filassi

**Affiliations:** I Setor de Mastologia, Disciplina de Ginecologia, Departamento de Ginecologia e Obstetricia, Hospital das Clínicas HCFMUSP, Faculdade de Medicina, Universidade de Sao Paulo, Sao Paulo, SP, BR; II Disciplina de Ginecologia, Departamento Ginecologia e Obstetricia, Hospital das Clinicas HCFMUSP, Faculdade de Medicina, Universidade de Sao Paulo, Sao Paulo, SP, BR

## INTRODUCTION AND PRESENTATION OF THE ETHICAL ISSUE

Breast cancer (BC) is the most common type of cancer in Brazil; it was responsible for 15,593 deaths in 2015, and an estimated 59,700 new cases were diagnosed in 2018 (1). BC in Brazil has some peculiarities when compared to other countries; 35 to 41.1% of all cases are diagnosed among patients younger than 50 years old, and most of the operable cases are diagnosed in later stages (53.5% stage 2 and 23.2% stage 3) (2), despite the opportunistic mammographic screening program (MSP) supported by the national government. The screening model adopted in Brazil recommends a mammogram every two years for women between the ages of 50 and 69 (3). However, the frequent diagnosis of BC in women at younger ages in Brazil leads to the claim that over 40% of the women diagnosed with cancer were not eligible for the MSP to begin with. The late stage at diagnosis, on the other hand, leads to the hypothesis that the current screening program is either not effective or individuals do not have proper access to it. Additionally, BC mortality in Brazil has been increasing in the last decades (4). Together, these factors pose an ethical issue, as investing valuable resources in an ineffective program negatively impacts the whole society and, therefore, smarter resource allocation schemes should be implemented to address this issue. In this editorial, we will dispute, from a utilitarian approach, the usefulness of the current MSP recommendation in a country with great inequalities among its regions and will also discuss an alternative to breast cancer screening from a Rawlsian perspective.

## RECOMMENDATION

In light of the ineffectiveness of the current Brazilian BC screening program in reducing mortality, we recommend resource reallocation to improve access to care and to establish a rapid pathway for diagnosis and treatment based on the hierarchical flow proposed by Migowski et al. (5). This algorithm proposes three distinct actions: increase educational activities in primary healthcare units to raise awareness and disseminate information about BC; offer shared decision-making regarding mammographic screening to asymptomatic patients between 50 and 69 years old in their primary care visit with a nurse or physician; and promote priority care, without the need to schedule appointments, for symptomatic patients, in which patients with suspected breast cancer would have priority referral to breast diagnostic services for diagnostic confirmation.

## SUPPORTING EVIDENCE AND ETHICAL THEORIES

Brazil is a developing country with a population of 209.3 million inhabitants that has large economic and social disparities between its 5 regions (6). The country also presents inequalities in the distribution of health and human resources, with significant variation in the number of cancer care hospitals and physicians across its regions (4). There are also inequalities in health outcomes in the country that might be explained by the duality of its health system. Healthcare in Brazil can be provided via the public system (Sistema Único de Saúde – SUS) or via the private system (based on health insurance plans or self-paying individuals). This system duality is somewhat perverse as it perpetuates the idea that a small part of the population can afford state-of-the-art private healthcare, whereas the majority, approximately 71% of the population, has to rely solely on the SUS. When we compare patients from these two settings, we can see a striking difference; the majority of patients in the private setting are diagnosed with early stage disease, while most patients on SUS are diagnosed with locally advanced BC, clearly denoting that the lack of access is the main obstacle to early detection (7). The perversity comes mostly from the fact that the SUS has long waiting lines for referrals; for example, the mean time between BC presentation and biopsy in patients on SUS is between 75-185 days, which might impact prognosis (7); even after diagnosis, there are still waiting lines for treatment (the average time for radiation therapy is approximately 113.4 days (8)). In the private system, for comparison purposes, a patient can obtain a mammogram, have a biopsy and start treatment in less than 30 days. In addition, SUS does not cover all cancer drugs that are readily available in the private setting. Taken together, all these factors lead to a great difference in health outcomes, favoring patients from the private setting, as demonstrated by Lee et al. (9). A large body of evidence corroborates this argument, supporting that the existence of healthcare access barriers and socioeconomic inequalities are the main reasons for late-stage diagnosis, rather than a lack of screening (9-12).

In a country with the above-demonstrated inequalities, the recommendation proposed in this paper is aligned with Rawls’ two principles of justice: the first is that each person has the right to equal basic rights and liberties, and the second is that social and economic inequalities are acceptable as long as they benefit the least advantaged members of society (13). The first part of the recommendation, which promotes educational sessions, is a clear example of Rawls’ first principle of justice. The promotion of education to raise awareness of an increasingly important health issue in the least educated part of the population provides equal access to a basic right (education itself). The second part of the recommendation can be justified by Rawls’ second principle of justice. By providing easy and fast access to patients with symptomatic breast conditions, removing the necessity to schedule appointments and prioritizing referrals, we are allocating resources to remove barriers to accessing the healthcare system, with the objective of reducing delays in diagnosis and treatment, and therefore, reducing inequalities and favoring the least advantaged members of society that do not have access to private healthcare. Although the recommendation favors the least advantaged, it does not infringe on the rights of other individuals, as asymptomatic patients would still have the right to undergo mammographic screening after counseling with their primary care physicians. Furthermore, the proposed recommendation removes age boundaries, promoting equal access to the breast cancer care continuum to women younger than 50 years old, an unmet need of the current adopted model since these patients, who account for up to 41.1% of all BC cases (2), do not fulfill the age requirement for the government’s MSP.

One might argue that the resource reallocation proposed here will decrease the odds of early diagnosis in the most-favored, well-educated portion of the population. We can refute this claim by quoting Rawls himself and acknowledging that &quot;All social primary goods are to be distributed equally unless an unequal distribution of any of all of these goods is to the advantage of the least favored&quot; (13). Moreover, the most favored, well-educated part of the population would not be forbidden to have screening exams; we can argue that the wealthy population can utilize their private insurance and conduct the screening on their own without the need for public resources.

However, let’s hypothesize for a moment that Brazil does not have any barriers to accessing healthcare. Would the current MSP then be the ideal intervention in the breast cancer care continuum? The answer would still be no. The evidence supporting the MSP is not unanimous, and large recently published clinical trials have failed to demonstrate a reduction in BC mortality from screening (14,15). Even if those studies turned out to show a BC mortality reduction, would their results be applicable to Brazil’s reality? Again, the answer would be no. Those studies were carried out in countries with high human development indexes (an index that assesses the health, education and economy of countries) and in the setting of organized screening programs. Brazil not only has a much lower human development index but also promotes opportunistic screening due to a weak organizational structure, instead of the organized modalities evaluated in the referenced studies. In this way, the data from those studies should not be applied in Brazil to justify the adoption of an MSP. A local manuscript recently published by Vale et al suggested that the opportunistic screening program used in Brazil promoted downstaging in BC in the most developed state in the country; however, it did not show BC mortality reduction, which is the ultimate goal of any cancer screening program (16). Those are strong arguments to support, from a utilitarian perspective, that screening by itself is not an ideal intervention in Brazil as there is not enough evidence to support that it provides maximal benefit to society. Moreover, mammographic screening has harmful potential, having its own associated mortality and morbidity risks, including breast radiation, anxiety, unnecessary biopsies (due to false-positive mammogram results) and risks involved in diagnostic surgery (17).

One might argue, however, that having a mammogram once a year is not a costly intervention. However, mammographic screening of an asymptomatic population is indeed a costly intervention, as it does not include only the cost of the initial mammogram. Ten percent of patients get called back for additional imaging, and 10% of those will have a biopsy. Only 30% of all biopsies will be positive, meaning that 70% of biopsied patients will receive a biopsy for normal breast tissue (18). With such high levels of false-positive results, screening in this setting cannot be justified from a utilitarian point of view.

## CONCLUSION

When we weigh the benefits and harms of the current MSP in Brazil, in the context of increasing breast cancer mortality in recent decades, it is very difficult to justify the resources needed to promote this intervention in the breast cancer care continuum from a utilitarian perspective. An alternative approach promoting easy and fast healthcare access for symptomatic patients and relegating the MSP to a secondary role favors a vulnerable portion of the Brazilian population who rely solely on the public system. In this way, allocating more resources to favor the least advantaged members of society is not only ethically acceptable, but also a way of promoting justice.
